# Supplementary material for: Irisin Attenuates Muscle Impairment during Bed Rest through Muscle-Adipose Tissue Crosstalk
Source: Biology (Basel). 2022 Jun 30;11(7):999. doi: 10.3390/biology11070999 (PMC9311907; doi:10.3390/biology11070999)
Supplement: Supplementary file 1 [file biology-11-00999-s001.zip › biology-1777951-supplementary.pdf]

biology

MDPI

Article

Irisin attenuates muscle impairment during bed rest through muscle-adipose tissue crosstalk

Andrea D'Amuri [1*], Juana Maria Sanz [2*], Stefano Lazzer [3], Rado Pišot [4], Bostjan Šimunič [4], Gianni Biolo [5], Giovanni Zuliani [1,6], Mladen Gasparini [7], Marco Narici [8], Bruno Grassi [3], Carlo Reggiani [8], Edoardo Dalla Nora [1], and Angelina Passaro [1,6,9**]**

[1] Medical Department, University Hospital of Ferrara Arcispedale Sant'Anna, Via A. Moro 8, I-44124, Ferrara, Italy; dmrndr@unife.it (A.D.); giovanni.zuliani@unife.it (G.Z.); edoardo.dallanora@unife.it (E.D.N.); angelina.passaro@unife.it (A.P.).
[2] Department of Chemical, Pharmaceutical and Agricultural Sciences, University of Ferrara, Via Luigi Borsari 46, 44121 Ferrara, Italy; juana.sanz@unife.it (J.M.S.).
[3] Department of Medicine, University of Udine, Piazzale M. Kolbe 4, I-33100, Udine, Italy; stefano.lazzer@uniud.it (S.L.); bruno.grassi@uniud.it (B.G.).
[4] Institute for Kinesiology Research, Science and Research Centre Koper, Garibaldijeva 1, SI-6000 Koper, Slovenia; Rado.Pisot@zrs-kp.si (R.P.); Bostjan.Simunic@zrs-kp.si (B.S.).
[5] Department of Medicine, Surgery and Health Sciences, University of Trieste, Strada di Fiume, 447, I-340149, Trieste, Italy; biolo@units.it (G.B.).
[6] Department of Translational Medicine, University of Ferrara - Via Luigi Borsari, 46 – I-44121, Ferrara, Italy; giovanni.zuliani@unife.it (G.Z.); angelina.passaro@unife.it (A.P.).
[7] Department of Vascular Surgery, Izola General Hospital, Polje 40, SI-6310, Izola-Isola, Slovenia; Mladen.gasparini@sb-izola.si (M.G.).
[8] Department of Biomedical Sciences, University of Padua, via Marzolo 3, I-35131, Padua, Italy; marco.narici@unipd.it (M.N.); Carlo.reggiani@unipd.it (C.R.).
[9] Research and Innovation Section, University Hospital of Ferrara Arcispedale Sant'Anna, Via A. Moro 8, I-44124, Ferrara, Italy; angelina.passaro@unife.it (A.P.).

* These authors, andrea.damuri@unife.it; juana.sanz@unife.it contributed equally and share first author-ship
** Correspondence: EDN: edoardo.dallanora@unife.it; A.P.: angelina.passaro@unife.it.

Citation: Lastname, F.; Lastname, F.; Lastname, F. Title. *Biology* **2022**, *11*, 999. https://doi.org/10.3390/biology11070999

Academic Editor: Firstname Lastname

Received: date
Accepted: date
Published: date

Publisher's Note: MDPI stays neutral with regard to jurisdictional claims in published maps and institutional affiliations.

Copyright: © 2022 by the authors. Submitted for possible open access publication under the terms and conditions of the Creative Commons Attribution (CC BY) license (https://creativecommons.org/licenses/by/4.0/).

It contains supplementary data, useful for understanding the main text (the main text contains them).

Table S1. Effect of 14-day Bed Rest on the body composition and muscle parameters.

Outcome measures	Bed Rest Time		Bed Rest Effect		
	BDC<br>Mean±SD	BR14<br>Mean±SD	Change within group<br>MD (95% CI)	P_{value}	Partial η2
**BMI (kg/mq)**	25.8±4.0	24.9±3.8	-0.9 (-1.1–-0.7)	**0.000**	0.822
**FM (kg)**	16.9±6.3	18.2±7.1	1.4 (0.3 – 2.4)	**0.013**	0.248
**FM (%)**	21.2±6.2	23.5±7.0	2.3 (1.0 – 3.7)	**0.002**	0.362
**FFM (kg)**	61.5±7.8	57.4±6.3	-4.1 (-5.4 – -2.7)	**0.000**	0.636
**FFM (%)**	78.8±6.2	76.5±7.0	-2.3 (-3.7 – -1.0)	**0.002**	0.362
**BCM (kg)**	33.0±5.1	30.8±3.7	-2.2 (-3.3 – -1.0)	**0.001**	0.423
**MM (kg)**	40.6±6.0	38.0±4.4	-2.7 (-4.0 – -1.4)	**0.000**	0.462
**QMV (cm^3)**	1775.6±280.4	1641.7±257.8	-133.9 (-165.8 – -102.0)	**0.000**	0.775
**MEP (W)**	2946.3±715.1	2553.7±728.1	-392.5 (-514.4 – -270.6)	**0.000**	0.670
**Specific MEP (W cm^{-3})**	1.65±0.26	1.55±0.30	-0.11 (-0.19 – -0.02)	**0.015**	0.242
**MVC (N)**	587.0±126.6	518.0±115.5	-69.0 (-100.4 – -37.7)	**0.000**	0.487
**Specific MVC (N cm^{-3})**	0.33±0.08	0.32±0.06	-0.02 (-0.04 – 0.00)	0.076	0.136
**CSA Fiber Slow/1 (μm^2)**	6082.9±2668.3	5291.2±2537.8	-791.7 (-1808.7 – 225.3)	0.120	0.111
**CSA Fiber Fast 2 (μm2)**	6081.3±2534.8	5259.4±2402.9	-821.9 (-1654.4 – 10.7)	0.053	0.160
**F_0 Fiber Slow/1 (mN)**	0.739±0.308	0.470±0.234	-0.269 (-0.416 – -0.122)	**0.001**	0.422
**F_0 Fiber Fast 2 (mN)**	0.786±0.467	0.567±0.286	-0.219 (-0.392 – -0.045)	**0.016**	0.256
**P_0 Fiber Slow/1 (mN mm^{-2})**	138.5±63.6	94.0±33.9	-44.5 (-83.8 – -5.3)	**0.028**	0.219
**P_0 Fiber Fast 2 (mN mm^{-2})**	138.3±45.2	118.2±44.9	-20.1 (-54.7 – 14.5)	0.240	0.065
**V_0 Fiber Slow/1 (L s^{-1})**	0.572±0.344	0.161±0.146	-0.411 (-0.611 – -0.210)	**0.001**	0.559
**V_0 Fiber Fast 2 (L s^{-1})**	2.051±0.817	0.659±0.442	-1.392 (-1.897 – -0.887)	**0.000**	0.697

*BDC, baseline data collection; BR14, after 14-day bed rest data collection; BMI, body mass index; FM, fat mass; FFM, free fat mass; BCM, body cellular mass; MM, muscle mass; QMV, quadriceps muscular volume; MEP, maximal explosive power; Specific MEP, ratio between MEP and QMV; MVC, maximal voluntary contraction; Specific MVC, ratio between MVC and QMV; CSA, cross sectional area; F0, isometric force; P0, specific force; V0, unloaded shortening velocity. Analysis performed with General Linear Model (GLM) Repeated Measures, Within-Subjects. Mean ± standard deviation (SD); median deviation (MD) and 95% confidence intervals (95% CI).

Table S2. Correlation between Irisin (BDC, BR14, and Irisin variation) and body composition, muscle performance and fiber types properties.

	Irisin (mg/L)							
	**BDC**		**BR14**		**$\Delta_{\%}$ BR14 vs BDC**		**Δ BR14 vs BDC**	
	correlation coefficient (r)	P_{value}	correlation coefficient (r)	P_{value}	correlation coefficient (r)	P_{value}	correlation coefficient (r)	P_{value}
**Age**	0.084	0.704	0.133	0.545	0.092	0.676	0.089	0.687

* BDC, baseline data collection; BR14, after 14-day Bed Rest data collection; Δ%variable, percentage difference of the variables of interest between BR14 and BDC; Δvariable, difference of the variables of interest between BR14 and BDC.

Table S3. Effect of 14-day Bed Rest and age and cognitive intervation group on irisin.

		Bed Rest Time		Bed Rest Effect			Bed Rest x Group effect		
**Outcome measures**	**Group**	**BDC** Mean±SD	**BR14** Mean±SD	Change within group MD (95% CI)	P_{value}	Partial η2	Change between group MD (95% CI)	P_{value}	Partial η2
**Irisin (mg/L)**	Young adults	4.58±1.13	6.07±2.65	1.77 (0.45 – 3.09)	0.011	0.271	1.49 (-0.78 – 3.77)	0.665	0.009
	Older adults	5.01±3.20	7.06±3.91				2.05 (0.49 – 3.60)		
	noCCT-Older	4.29±0.97	6.83±3.79	2.05 (0.45 – 3.64)	0.016	0.351	2.53 (-0.33 – 5.40)	0.524	0.030
	CCT-Older	5.72±4.46	7.28±4.28				1.56 (-0.48 – 3.60)		

* BDC, baseline data collection; BR14, after 14-day bed rest data collection; FM, fat mass; FFM, free fat mass. Young adults: number 7 subjects; Older adult: number 16 subjects. Analysis performed with General Linear Model (GLM) Repeated Measures, Within-Subjects and Between-Subjects test. Mean ± standard deviation (SD); median deviation (MD) and 95% confidence intervals (95% CI).

Table S4. Correlation between Irisin (BDC, BR14, and Irisin variation) and body composition, muscle performance and fiber types properties.

	Irisin							
	**BDC Irisin vs BDC variable**		**BR14 Irisin vs BR14 variable**		**$\Delta_{\%}$Irisin vs $\Delta_{\%}$variable**		**ΔIrisin vs Δvariable**	
	correlation coefficient (r)	P_{value}	correlation coefficient (r)	P_{value}	correlation coefficient (r)	P_{value}	correlation coefficient (r)	P_{value}
**BMI (kg/mq)**	0.524	**0.010**	0.360	0.091	0.012	0.955	0.058	0.791
**FM (kg)**	0.296	0.170	0.519	**0.011**	-0.208	0.340	-0.167	0.448
**FFM (kg)**	0.508	**0.013**	0.085	0.700	0.109	0.621	0.143	0.515
**BCM (kg)**	0.547	**0.007**	0.118	0.592	0.203	0.353	0.232	0.263
**MM (kg)**	0.556	**0.006**	0.117	0.596	0.192	0.381	0.232	0.286
**QMV (cm^3)**	0.332	0.122	0.107	0.628	0.146	0.507	0.287	0.184
**MEP (W)**	0.216	0.322	0.025	0.910	-0.067	0.760	-0.042	0.849
**Specific MEP (W cm^{-3})**	0.048	0.829	0.013	0.952	-0.116	0.598	-0.142	0.518
**MVC (N)**	0.190	0.385	-0.093	0.672	0.425	**0.043**	0.476	**0.022**
**Specific MVC (N cm^{-3})**	0.087	0.692	-0.022	0.919	0.375	0.078	0.369	0.083
**CSA Fiber Slow/1 (μm^2)**	-0.022	0.922	-0.131	0.550	-0.559	**0.007**	-0.614	**0.002**
**CSA Fiber Fast/2 (μm^2)**	0.076	0.732	-0.035	0.873	-0.270	0.214	-0.395	0.062
**F_0 Fiber Slow/1 (mN)**	0.047	0.839	-0.086	0.704	0.110	0.636	0.097	0.677
**F_0 Fiber Fast/2 (mN)**	0.140	0.535	-0.047	0.841	0.199	0.386	0.276	0.213
**P_0 Fiber Slow/1 (mN mm^{-2})**	0.181	0.433	0.060	0.792	0.473	**0.030**	0.346	0.124
**P_0 Fiber Fast/2 (mN mm^{-2})**	0.186	0.407	-0.087	0.699	0.215	0.337	0.240	0.295
**V_0 Fiber Slow/1 (L s^{-1})**	-0.317	0.162	0.421	0.087	-0.191	0.479	-0.076	0.779
**V_0 Fiber Fast/2 (L s^{-1})**	0.104	0.691	-0.535	0.059	-0.370	0.171	-0.267	0.318

* BDC, baseline data collection; BR14, after 14-day bed rest data collection; FM, fat mass; FFM, free fat mass. Young adults: number 7 subjects; Older adult: number 16 subjects. Analysis performed with General Linear Model (GLM) Repeated Measures, Within-Subjects and Between-Subjects test. Mean ± standard deviation (SD); median deviation (MD) and 95% confidence intervals (95% CI).

Table S5. Effect of 14-day Bed Rest and tertile BDC Irisin on the body composition and muscle parameters.

Outcome measures	Group Tertiles Irisin BDC	Bed Rest Time BDC Mean±SD	Bed Rest Time BR14 Mean±SD	Bed Rest Effect: Change within group MD (95% CI)	P_{value}	Partial η2	Bed Rest x Group effect: Change between group MD (95% CI)	P_{value}	Partial η2
	1sttertile	60.7±8.1	58.1±6.8				-2.6 (-5.4 – 0.2)		
**FFM (kg)**	2ndtertile	60.7±6.4	56.9±6.6	-4.1 (-5.4 – -2.8)	**<0.001**	0.680	-3.9 (-5.7 – -2.1)	0.134	0.182
	3rdtertile	63.0±9.3	57.3±6.4				-5.7 (-8.5 – -2.9)		
	1sttertile	39.5±5.1	37.8±4.5				-1.7 (-4.3 – 0.8)		
**MM (kg)**	2ndtertile	40.1±5.6	37.5±4.7	-2.7 (-4.0– -1.4)	**<0.001**	0.483	-2.6 (-5.0 – -0.2)	0.405	0.086
	3rdtertile	42.3±7.4	38.6±4.5				-3.7 (-6.4 – -1.1)		
	1sttertile	1743.9±226.7	1611.4±222.4				-132.5 (-190.3 – -74.7)		
**QMV (cm^3)**	2ndtertile	1739.1±199.6	1604.0±232.5	-133.9 (-167.6 – -100.2)	**<0.0001**	0.774	-135.1 (-199.1 – -71.1)	0.998	0.000
	3rdtertile	1839.3±393.0	1705.1±325.6				-134.1 (-209.9 – -58.3)		
	1sttertile	2766.3±749.7	2342.8±648.5				-423.5 (-548.3 – -298.7)		
**MEP (W)**	2ndtertile	2904.1±692.4	2586.6±665.8	-389.4 (-516.2 – -262.6)	**<0.001**	0.672	-317.6 (-564.2 – -70.9)	0.720	0.032
	3rdtertile	3163.1±736.0	2736.0±880.8				-427.1 (-760.0 – -94.3)		
	1sttertile	0.32±0.07	0.31±0.07				-0.13 (-0.20– -0.06		
**Specific MEP ($W\ cm^{-3}$)**	2ndtertile	0.36±0.08	0.36±0.05	-0.017 (-0.036– 0.003)	0.091	0.136	-0.05 (-0.21 – 0.11)	0.533	0.061
	3rdtertile	0.33±0.08	0.30±0.06				-0.13 (-0.37 – 0.10)		
	1sttertile	554.2±125.6	492.0±132.6				-62.3 (-113.3 – -11.2)		
**MVC (N)**	2ndtertile	621.9±115.9	569.3±100.4	-68.4 (-100.6– -36.1)	**<0.001**	0.494	-52.6 (-131.9 – 26.7	0.594	0.051
	3rdtertile	589.3±143.6	499.0±108.9				-90.3 (-152.9 – -27.6)		
	1sttertile	1.58±0.32	1.45±0.29				-0.01 (-0.05 – 0.02)		
**Specific MVC ($N\ cm^{-3}$)**	2ndtertile	1.66±0.27	1.61±0.30	-0.10 (-0.19 – -0.02)	**<0.020**	0.241	-0.01 (-0.05 – 0.04)	0.657	0.041
	3rdtertile	1.72±0.18	1.59±0.33				-0.03 (-0.07 – 0.00)		

BDC, baseline data collection; BR14, after 14-day Bed Rest data collection; FFM, free fat mass; MM, muscle mass; QMV, quadriceps muscle volume; MEP, maximal explosive power of lower limb; Specific MEP, ratio between MEP and QMV; MVC, maximal voluntary contraction; Specific MVC, ratio between MVC and QMV; 1sttertile, 1sttertile Irisin BDC (n°8 subjects); 2ndtertile, 2ndtertile Irisin BDC (n° 7 subjects); 3rd tertile, 3rd tertile Irisin BDC (n°8 subjects).

Analysis performed with General Linear Model (GLM) Repeated Measures, Within-Subjects and Between-Subjects test. Mean ± standard deviation (SD); median deviation (MD) and 95% confidence intervals (95% CI).

Table S6. Effect of 14-day Bed Rest and tertile Irisin BDC group effect on the fiber type properties.

Outcome measures	Group Tertile Irisin BDC	Bed Rest Time		Bed Rest Effect			Bed Rest x Group effect		
		**BDC** Mean±SD	**BR14** Mean±SD	Change within group MD (95% CI)	P_{value}	Partial η2	Change between group MD (95% CI)	P_{value}	Partial η2
**Fiber Slow/1 (%)**	1st tertile	41.79±24.12	38.88±27.19				-2.91 (-21.15 – 15.32)		
	2nd tertile	39.20±13.07	33.72±9.08	-3.44 (-14.68 – 7.80)	0.530	0.020	-5.48 (-23.82 – 12.86)	0.964	0.004
	3rd tertile	31.59±21.26	29.66±20.98				-1.93 (-29.50 – 25.63)		
**Fiber Fast/2 (%)**	1st tertile	58.21±24.12	70.40±12.60				12.19 (0.19 – 24.19)		
	2nd tertile	60.80±13.07	66.97±9.13	7.72 (-2.15 – 17.60)	0.118	0.117	6.17 (-11.31 – 23.64)	0.791	0.023
	3rd tertile	68.41±21.26	73.22±15.77				4.81 (-21.01 – 30.64)		
**CSA Fiber Slow/1 (μm^2)**	1st tertile	4565.5±1514.0	4340.9±1481.5				-224.7 (-1659.1 – 1209.8)		
	2nd tertile	6677.3±2657.5	4614.9±1381.0	-818.7 (-1812.7 – 175.3)	0.101	0.135	-2062.3 (-4328.7 – 204.1)	0.213	0.150
	3rd tertile	7222.7±3191.3	7053.7±3565.3				-169.0 (-2473.8 – 2135.7)		
**CSA Fiber Fast/2 (μm^2)**	1st tertile	4926.4±1724.0	4677.0±1380.9				-249.4 (-2111.9 – 1613.0)		
	2nd tertile	5621.8±661.0	4236.6±886.4	-845.335 (-1698.529 – 7.859)	**0.052**	0.176	-1385.2 (-2645.7 – -124.7)	0.539	0.060
	3rd tertile	7638.1±3513.0	6736.8±3411.5				-901.4 (-2654.0 – 851.3)		
**F_0 Fiber Slow/1 (mN)**	1st tertile	0.713±0.137	0.713±0.137				-0.274 (-0.571 – 0.023)		
	2nd tertile	0.621±0.257	0.371±0.152	-0.269 (-0.425 – -0.113)	**0.002**	0.422	-0.250 (-0.515 – 0.014)	0.983	0.002
	3rd tertile	0.884±0.436	0.600±0.264				-0.283 (-0.656 – 0.090)		
**F_0 Fiber Fast/2 (mN)**	1st tertile	0.590±0.208	0.590±0.208				-0.070 (-0.361 – 0.221)		
	2nd tertile	0.773±0.214	0.493±0.218	-0.209 (-0.388 – -0.029)	**0.025**	0.249	-0.280 (-0.625 – 0.065	0.554	0.063
	3rd tertile	0.944±0.701	0.668±0.370				-0.276 (-0.664 – 0.112)		
**P_0 Fiber Slow/1 ($mN\ mm^{-2}$)**	1st tertile	177.3±71.9	101.4±45.2				-75.9 (-177.4 – 25.6)		
	2nd tertile	103.5±47.2	89.6±34.7	-44.5 (-84.3 – -4.8)	**0.030**	0.235	-13.9 (-86.2 – 58.3)	0.426	0.090
	3rd tertile	134.7±53.9	91.0±22.2				-43.8 (-105.1 – 17.6)		
**P_0 Fiber Fast/2 ($mN\ mm^{-2}$)**	1st tertile	142.3±35.9	128.2±53.4				-14.1 (-89.4 – 61.1)		
	2nd tertile	150.1±54.0	128.7±52.2	-19.9 (-56.5 – 16.7)	0.269	0.064	-21.3 (-110.0 – 67.3)	0.970	0.003
	3rd tertile	124.5±46.5	100.2±26.4				-24.3 (-81.3 – 32.7)		
**V_0 Fiber Slow/1 ($L\ s^{-1}$)**	1st tertile	0.608±0.450	0.062±0.042				-0.547 (-1.081 – -0.012)		
	2nd tertile	0.522±0.327	0.178±0.169	-0.416 (-0.633 – -0.199)	**0.001**	0.569	-0.344 (-0.707 – 0.019)	0.652	0.064
	3rd tertile	0.257±0.133	0.257±0.133				-0.357 (-0.879 –0.164)		
**V_0 Fiber Fast/2 ($L\ s^{-1}$)**	1st tertile	1.926±0.547	0.926±0.587				-1.001 (-1.916 – -0.085)		
	2nd tertile	1.925±0.905	0.572±0.228	-1.394 (-1.910 – -0.879)	**<0.001**	0.724	-1.353 (-2.245 – -0.462)	0.410	0.128
	3rd tertile	0.498±0.436	0.498±0.436				-1.829 (-3.345 – -0.312)		

BDC, baseline data collection; BR14, after 14-day Bed Rest data collection; CSA, cross sectional area; F0, isometric force; P0, specific force; V0, unloaded shortening velocity. 1st tertile, 1st tertile Irisin BDC (n°8 subjects); 2nd tertile, 2nd tertile Irisin BDC (n° 7 subjects); 3rd tertile, 3rd tertile Irisin BDC (n°8 subjects). Analysis performed with General Linear Model (GLM) Repeated Measures, Within-Subjects and Between-Subjects test. Mean ± standard deviation (SD); median deviation (MD) and 95% confidence intervals (95% CI).

Table S7. Effect of Bed Rest and tertile %Irisin group effect on body composition and muscle parameters.

Outcome measures	Group<br>Tertile Δ%Irisin	Bed Rest Time<br>BDC<br>Mean±SD	 BR14<br>Mean±SD	Bed Rest Effect<br>Change within group<br>MD (95% CI)	 P_{value}	 Partial η2	Bed Rest x Group effect<br>Change between group<br>MD (95% CI)	 P_{value}	 Partial η2
	1st tertile	63.4±10.1	58.2±7..2				-5.2 (-8.4 – -2.1)		
**FFM (kg)**	2nd tertile	66.6±8.4	57.6±7.8	-4.028 (-5.4 – -2.7)	**<0.001**	0.652	-3.0 (-6.4 – 0.5	0.389	0.090
	3rd tertile	60.4±4.8	56.5±4.7				-3.9 (-5.1 – -2.7)		
	1st tertile	43.6±7.2	39.5±4.9				-4.1 (-6.9 – -1.3)		
**MM (kg)**	2nd tertile	37.7±6.2	36.3±5.3	-2.6 (-3.9 – -1.4)	**<0.001**	0.489	-1.4 (-4.4 – 1.6)	0.219	0.141
	3rd tertile	40.3±3.1	37.9±2.6				-2.4 (-4.0 – -0.9)		
	1st tertile	1869.0±364.9	1724.6±312.3				-144.4 (-220.6 – -68.1)		
**QMV (cm3)**	2nd tertile	1648.0±233.3	1501.1±220.5	-134.4 (-167.3 – -101.5)	**<0.001**	0.784	-146.9 (-230.1 – -63.6)	0.604	0.049
	3rd tertile	1793.9±201.6	1681.9±200.5				-112.0 (-138.5 – -85.5)		
	1st tertile	3145.1±837.4	2848.1±817.0				-297.0 (-484.0 – -110.0)		
**MEP (W)**	2nd tertile	2588.9±572.4	2068.4±499.0	-397.9 (-519.5 – -276.2)	**<0.001**	0.699	-520.4 (-858.8 – -182.1)	0.317	0.108
	3rd tertile	3060.1±661.4	2684.0±656.7				-376.1 (-577.5 – -174.8)		
	1st tertile	1.67±0.25	1.64±0.28				-0.03 (-0.16 –0.09)		
**Specific MEP (W cm^{-3})**	2nd tertile	1.58±0.29	1.39±0.32	-0.11 (-0.19 – -0.03)	**0.012**	0.274	-0.18 (-0.45 – 0.08)	0.337	0.103
	3rd tertile	1.70±0.27	1.59±0.30				-0.11 (-0.21 – -0.01)		
	1st tertile	659.8±108.1	547.3±91.5				-112.5 (-178.1 – -46.9)		
**MVC (N)**	2nd tertile	530.9±101.0	454.7±121.9	-69.3 (-96.9 – -41.7)	**<0.001**	0.579	-76.1 (-137.1 – -15.2)	**0.026**	0.305
	3rd tertile	563.4±141.7	544.0±122.6				-19.4 (-52.7 – 13.9)		
	1st tertile	0.36±0.07	0.32±0.05				-0.04 (-0.08 – 0.00)		
**Specific MVC (N cm^{-3})**	2nd tertile	0.33±0.07	0.30±0.07	-0.02 (-0.03 – 0.00)	0.055	0.172	-0.02 (-0.07 – 0.02)	0.079	0.225
	3rd tertile	0.32±0.09	0.33±0.08				0.01(-0.01 – 0.03)		

BDC, baseline data collection; BR14, after 14-day Bed Rest data collection; Δ%, percentage change toward the BDC; FFM, free fat mass; MM, muscle mass; QMV, quadriceps muscle volume; MEP, maximal explosive power of lower limb; Specific MEP, ratio between MEP and QMV; MVC, maximal voluntary contraction; Specific MVC, ratio between MVC and QMV. 1st tertile, 1st tertile Δ%Irisin (n°8 subjects); 2nd tertile, 2nd tertile Δ%Irisin (n° 7 subjects); 3rd tertile, 3rd tertile Δ%Irisin (n°8 subjects).

Analysis performed with General Linear Model (GLM) Repeated Measures, Within-Subjects and Between-Subjects test. Mean ± standard deviation (SD); median deviation (MD) and 95% confidence intervals (95% CI).

Table S8. Effect of Bed Rest and tertile $\Delta_{\%}$Irisin group effect on the fiber type properties.

Outcome measures	Group<br>Tertile $\Delta_{\%}$Irisin	Bed Rest Time<br>BDC<br>Mean±SD	 BR14<br>Mean±SD	Bed Rest Effect<br>Change within group MD (95% CI)	 P_{value}	 Partial η2	Bed Rest x Group effect<br>Change between group MD (95% CI)	 P_{value}	 Partial η2
	1[st]tertile	44.6±14.5	34.1±15.0				-10.5 (-31.7 – 10.7)		
**Fiber Slow/1 (%)**	2[nd]tertile	34.7±20.9	38.8±32.0	-3.0 (-14.0 – 7.9)	0.568	0.017	-4.1 (-17.6 – 25.8)	0.538	0.060
	3[rd]tertile	32.7±23.8	30.0±12.5				-2.7 (-24.5 – 19.1)		
	1[st]tertile	55.4±14.5	71.5±9.8				16.1 (-1.0 – 33.1)		
**Fiber Fast/2 (%)**	2[nd]tertile	65.3±20.9	69.1±17.5	-3.0 (-14.0 – 7.9)	0.568	0.017	3.8 (-13.6 – 21.2)	0.433	0.080
	3[rd]tertile	67.3±23.8	70.3±11.8				3.0 (-18.5 – 24.5)		
	1[st]tertile	5928.4±3003.4	6333.8±3412.4				405.5 (-559.5 – 1370.4)		
**CSA Fiber Slow/1 (μm²)**	2[nd]tertile	5133.4±1995.2	5175.3±2281.9	-848.7 (-1644.7 – -52.6)	**0.038**	0.208	42.0 (-1665.4 – 1749.4)	**0.003**	0.455
	3[rd]tertile	7209.1±2802.0	4215.6±966.5				-2993.4 (-5077.0 – -909.9)		
	1[st]tertile	5903.1±3227.9	5875.5±3716.6				-27.6 (-1592.2–1537.1)		
**CSA Fiber Fast/2 (μm²)**	2[nd]tertile	5067.2±1369.3	4782.6±1405.4	-799.5 (-1564.9–-34.1)	**0.041**	0.192	-284.7 (-1811.2–1241.9)	0.061	0.243
	3[rd]tertile	7146.7±2409.2	5060.5±1391.0				-2086.2 (-3528.1–-644.2)		
	1[st]tertile	0.812±0.436	0.517±0.302				-0.295 (-0.640– 0.050)		
**F_0 Fiber Slow/1 (mN)**	2[nd]tertile	0.732±0.149	0.466±0.256	-0.269 (-0.425–-0.113)	**0.002**	0.423	-0.265 (-0.582– 0.052)	0.966	0.004
	3[rd]tertile	0.674±0.305	0.427±0.143				-0.247 (-0.525– 0.030)		
	1[st]tertile	0.984±0.746	0.575±0.433				-0.409 (-0.900– 0.082)		
**F_0Fiber Fast/2 (mN)**	2[nd]tertile	0.592±0.206	0.498±0.244	-0.216 (-0.389–-0.043)	**0.017**	0.277	-0.093 (-0.423– 0.236)	0.274	0.134
	3[rd]tertile	0.758±0.208	0.612±0.156				-0.146 (-0.329 – 0.037)		
	1[st]tertile	142.5±40.8	85.6±44.4				-56.9 (-115.6–1.8)		
**P_0 Fiber Slow/1 (mN mm⁻²)**	2[nd]tertile	167.9±80.9	90.2±32.3	-44.5 (-82.8–-6.3)	**0.025**	0.249	-77.7 (-176.1–20.7)	0.216	0.156
	3[rd]tertile	105.3±54.5	106.3±23.4				1.0 (-68.0–70.0)		
	1[st]tertile	158.9±41.1	100.1±50.4				-58.8 (-134.8–17.3)		
**P_0Fiber Fast/2 (mN mm⁻²)**	2[nd]tertile	139.7±50.3	117.5±39.3	-21.8 (-55.4–11.8)	0.190	0.089	-22.2 (-94.7–50.4)	0.188	0.161
	3[rd]tertile	119.1±41.0	134.6±43.9				15.5 (-39.0–69.9)		
	1[st]tertile	0.603±0.469	0.176±0.212				-0.427 (-1.111– 0.256)		
**V_0Fiber Slow/1 (L s⁻¹)**	2[nd]tertile	0.460±0.127	0.172±0.120	-0.419 (-0.628–-0.209)	**0.001**	0.589	-0.288 (-0.486–-0.090)	0.568	0.083
	3[rd]tertile	0.674±0.411	0.133±0.125				-0.541 (-1.004–-0.078)		
	1[st]tertile	1.830±0.556	1.083±0.584				-0.747 (-1.335–-0.59)		
**V_0 Fiber Fast/2 (L s⁻¹)**	2[nd]tertile	2.326±0.890	0.516±0.355	-1.320 (-1.821–-0.820)	**<0.000**	0.714	-1.809 (-3.038–-0.181)	0.233	0.201
	3[rd]tertile	1.924±0.936	0.520±0.252				-1.404 (-2.283 –-0.525)		

BDC, baseline data collection; BR14, after 14-day Bed Rest data collection; Δ%, percentage change toward the BDC; CSA, cross sectional area; F0, isometric force; P0, specific force; V0, unloaded shortening velocity. 1st tertile, 1st tertile Δ%Irisin (n°8 subjects); 2nd tertile, 2nd tertile Δ%Irisin (n° 7 subjects); 3rd tertile, 3rd tertile Δ%Irisin (n°8 subjects). Analysis performed with General Linear Model (GLM) Repeated Measures, Within-Subjects and Between-Subjects test. Mean ± standard deviation (SD); median deviation (MD) and 95% confidence intervals (95% CI).

Table S9. Linear regression analysis model between dependent variable and ΔIrisin BR14 vs BDC (%).

Dependent Variable	Independent Variable	R^2	Standardized Coefficients β	ES	P_{value}
**$\Delta_{\%}$ MVC**	Δ% Irisin	0.181	0.425	0.037	**0.043**
**$\Delta_{\%}$ Specific MVC**	Δ% Irisin	0.156	0.394	0.000	0.063
**$\Delta_{\%}$ CSA Fiber Slow/1**	Δ% Irisin	0.355	-0.596	6.526	**0.003**
**$\Delta_{\%}$ CSA Fiber Slow/2**	Δ% Irisin	0.136	-0.369	6.328	0.083
**$\Delta_{\%}$ P_o Fiber Slow/1**	Δ% Irisin	0.224	0.473	0.247	**0.030**
**$\Delta_{\%}P_o$ Fiber Slow/2**	Δ% Irisin	0.046	0.215	0.206	0.337

* Δ% variable, Percentage difference of the variables of interest between BDC and BR14; Δ%Irisin, Percentage difference of Irisin between BDC and BR14; Δvariable, difference of the variables of interest between BR14 and BDC; MVC, maximal voluntary contraction; Specific MVC, ratio between MVC and QMV; CSA, cross sectional area; P_0, specific force.

Table S10. Multivariate stepwise linear regression analysis between Δ Irisin BR14 vs BDC (%) and possible independent predictors.

Dependent Variable	Independent Variable	R^2	StandardizedCoefficients β	ES	P_{value}
**$\Delta_{\%}$ Irisin**	**$\Delta_{\%}$CSA Fiber Slow/1**	0.300	0.548	0.359	0.010

* Δ%Irisin, Percentage difference of Irisin between BDC and BR14; Δ%variable, Percentage difference of the variables of interest between BDC and BR14. Model adjusted for Δ% MCV, Δ% P_0 Fiber Slow/1.

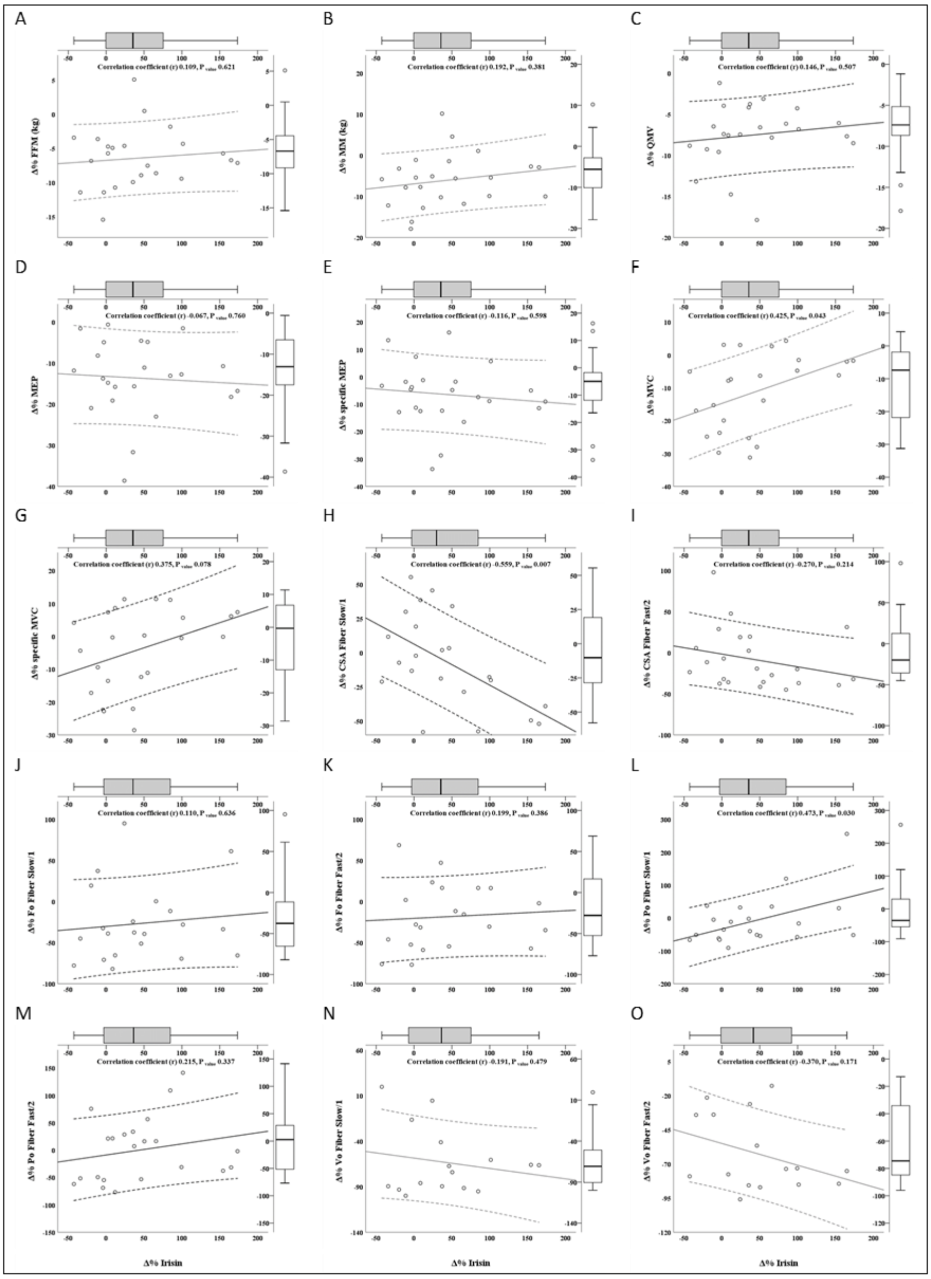

Figure S1. Correlation analysis between Irisin variation (percentage difference of the variables between BR14 and BDC) and body composition, muscle performance and fiber types properties variation (percentage difference of the variables between BR14 and BDC). In the different panels are described correlations between Δ Irisin (mg/L) BR14 vs BDC (%) and **panel A**: ΔFFM (%); **panel B**: ΔMM (%); **panel C**: ΔQMV (%); **panel D**: ΔMEP (%); **panel E**: Δspecific MEP (%); **panel F**: Δ MVC (%); **panel G**: Δ specific MVC (%); **panel H**: Δ CSA Fiber Slow/1(%); **panel I**: Δ CSA Fiber Fast/2(%); **panel J**: ΔF_0 Fiber Slow/1(%); **panel K**: ΔF_0 Fiber Fast/2(%); **panel L**: ΔP_0 Fiber Slow/1(%); **panel M**: ΔP_0 Fiber Fast/2(%); **panel N**: ΔV_0 Fiber Slow/1(%); **panel O**: ΔV_0 Fiber Fast/2(%). BDC, baseline data collection; BR14, after 14-day Bed Rest data collection; Δ% variable, percentage difference of the variables of interest between BR14 and BDC; FFM, free fat mass; MM, muscle mass; QMV, quadriceps muscular volume; MEP, maximal explosive power; Specific MEP, ratio between MEP and QMV; MVC, maximal voluntary contraction; Specific MVC, ratio between MVC and QMV; CSA, cross sectional area; F_0, isometric force; P_0, specific force; V_0, unloaded shortening velocity.

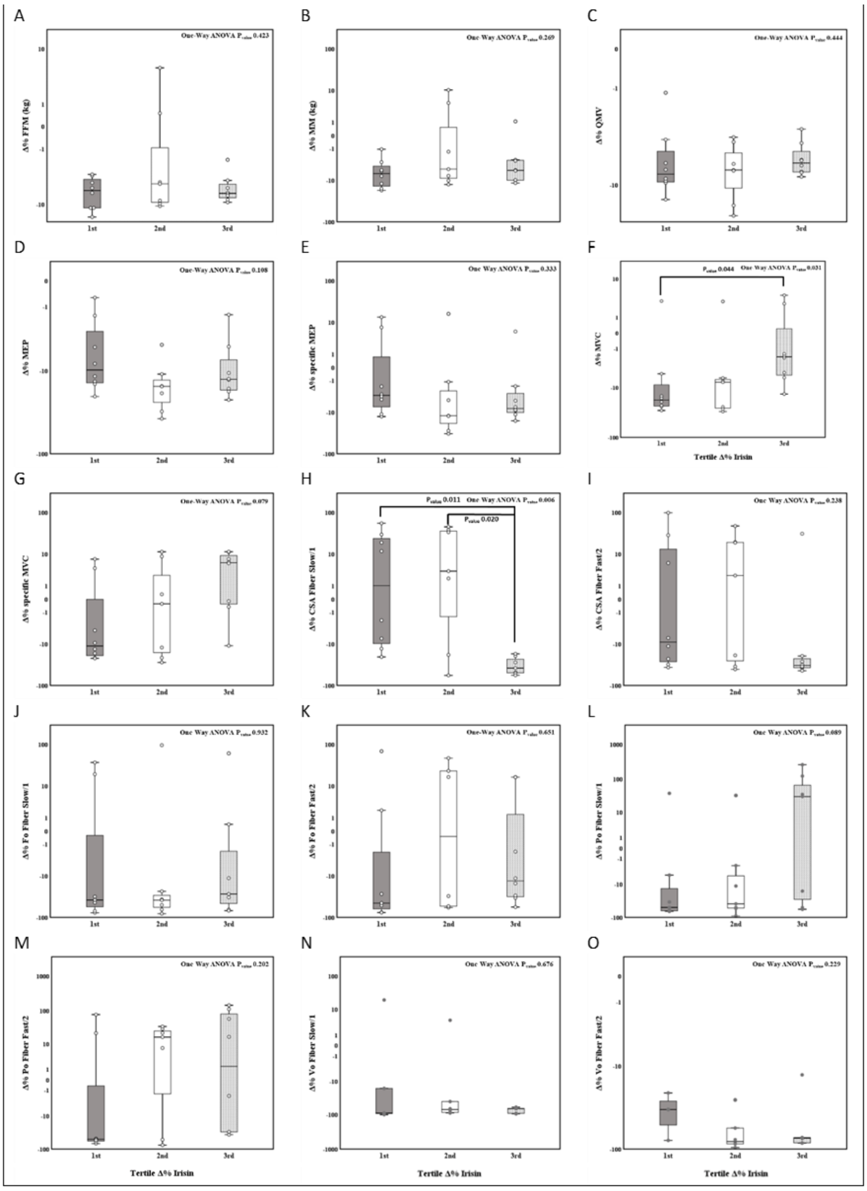

Figure S2. One-way analysis of variance for tertile Irisin variation (percentage difference of the variables between BR14 and BDC) and muscle performance variation (percentage difference of the variables between BR14 and BDC). In the different panels are described for tertile ΔIrisin (mg/L) BR14 vs BDC (%) the means (95% CI) of **panel A**: ΔFFM(%); **panel B**: ΔMM (%); **panel C**: ΔQMV(%); **panel D**: ΔMEP(%); **panel E**: Δspecific MEP(%); **panel F**: ΔMVC(%); **panel G**: Δspecific MVC(%); **panel H**: ΔCSA Fiber Slow/1(%); **panel I**: ΔCSA Fiber Fast/2(%); **panel J**: ΔF_0 Fiber Slow/1(%); **panel K**: ΔF_0 Fiber Fast/2(%); **panel L**: ΔP_0 Fiber Slow/1(%); **panel M**: ΔP_0 Fiber Fast/2(%); **panel N**: ΔV_0 Fiber Slow/1(%); **panel O**: ΔV_0 Fiber Fast/2(%). BDC, baseline data collection; BR14, after 14-day Bed Rest data collection; Δ% variable, percentage difference of the variables of interest between BR14 and BDC; FFM, free fat mass; MM, muscle mass; QMV, quadriceps muscular volume; MEP, maximal explosive power; Specific MEP, ratio between MEP and QMV; MVC, maximal voluntary contraction; Specific MVC, ratio between MVC and QMV; CSA, cross sectional area; F_0, isometric force; P_0, specific force; V_0, unloaded shortening velocity.
